# Introducing Short Reports—A new platform for swiftly communicating research findings

**DOI:** 10.1371/journal.ppat.1011734

**Published:** 2023-10-16

**Authors:** Julia Squarr

**Affiliations:** Public Library of Science, San Francisco, California, United States of America, Cambridge, United Kingdom, and Berlin, Germany; King’s College London, UNITED KINGDOM

We are excited to share that we are expanding *PLOS Pathogens’* offerings through the launch of a new manuscript type: **Short Reports**. This addition augments the spectrum of exceptional research we consider for publication.

Pathogens has long been esteemed for its rigorous long-form publications, presenting in-depth mechanisms alongside observations through our Research Articles. But, we recognize that impactful discoveries come in various sizes. The birth of Short Reports signifies our acknowledgment that sometimes exceptional content arrives in compact not-yet-fully understood form. Scientific findings vary—while some warrant patience until mechanistic intricacies are unraveled and verified by multiple experimental approaches, others are intrinsically groundbreaking, serving the scientific community best when shared in their nascent stages—even if the driving mechanism has not yet revealed itself—thus allowing for collective exploration. Furthermore, Short Reports find their niche by encapsulating succinct yet ingenious experiments. These studies might describe novel phenomena, reconcile erstwhile contradictory observations, untangle specific enigmas, or apply established techniques to furnish brief yet compelling responses to scientific queries.

We anticipate that Short Reports will provide a vital platform for researchers to swiftly communicate their findings.

Seizing this new article type, we aim to bolster impactful research within the scope of *PLOS Pathogens* embracing a wider array of research. Even as we do so, the principles that underpin our traditional Research Articles in discovery and translational science remain intact. Short Reports, just like its longer counterpart, are built upon the pillars of quality, scientific integrity, meticulous peer-review, and expert editorial oversight.

